# Stroke Recurrence and Pregnancy Outcomes in the Subsequent Pregnancies After Maternal Ischemic Stroke

**DOI:** 10.1161/STROKEAHA.125.051488

**Published:** 2025-09-19

**Authors:** Anna Richardt, Liisa Verho, Aino Korhonen, Kirsi Rantanen, Hannele Laivuori, Mika Gissler, Minna Tikkanen, Karoliina Aarnio, Petra Ijäs

**Affiliations:** Department of Neurology (A.R., A.K., K.R., K.A., P.I.), University of Helsinki and Helsinki University Hospital, Finland.; Department of Obstetrics and Gynecology (L.V., M.T.), University of Helsinki and Helsinki University Hospital, Finland.; Department of Medical and Clinical Genetics (H.L.), University of Helsinki and Helsinki University Hospital, Finland.; Institute for Molecular Medicine Finland, Helsinki Institute of Life Science, University of Helsinki, Finland (H.L.).; Department of Obstetrics and Gynecology, Tampere University Hospital, The Wellbeing Services County of Pirkanmaa, Finland (H.L.).; Center for Child, Adolescent and Maternal Health Research, Faculty of Medicine and Health Technology, Tampere University, Finland (H.L.).; Department of Data and Analytics, Finnish Institute for Health and Welfare, Helsinki, Finland (M.G.).; Academic Primary Health Care Centre, Region Stockholm, Sweden (M.G.).; Department of Molecular Medicine and Surgery, Karolinska Institute, Stockholm, Sweden (M.G.).

**Keywords:** ischemic stroke, pregnancy, postpartum period, secondary prevention

## Abstract

**BACKGROUND:**

Maternal ischemic stroke (IS) might affect the course of subsequent pregnancies. We aimed to study stroke recurrence, other complications, and the implementation of secondary prevention in subsequent pregnancies of women with a prior maternal IS.

**METHODS:**

Women diagnosed with IS during pregnancy or puerperium in Finland during the years 1987 to 2016, and the data of subsequent pregnancies were collected from the Medical Birth Register and Hospital Discharge Register. Diagnoses were verified from medical records. Three matched controls without a maternal stroke were identified for each case.

**RESULTS:**

Data on subsequent pregnancies were available for 90 patients with maternal IS after excluding patients who died within 1 year. Patients with maternal IS less frequently had at least 1 subsequent pregnancy (38.9% versus 51.7%; age-adjusted odds ratio, 0.55 [95% CI, 0.32–0.93]), and more frequently, multiple induced abortions (adjusted odds ratio, 6.24 [95% CI, 1.12–34.88]) than controls. Three women had a recurrent maternal IS or transient ischemic attack (8.6%). Patients with maternal IS more commonly had diabetes during pregnancy (29.1% versus 13.6%; adjusted odds ratio, 2.77 [95% CI, 1.17–6.59]) and hypertensive disorders of pregnancy than controls (12.7% versus 4.5%; adjusted odds ratio, 3.57 [95% CI, 1.02–12.51]). In the first subsequent pregnancy, perinatal deaths were more common in patients with maternal IS compared with controls (5.9% versus 0%; *P*=0.042). Most women used antithrombotic medication (87.9%) in the first subsequent pregnancy, but this declined in later pregnancies. The use of other secondary preventive medications was uncommon both before and during pregnancy.

**CONCLUSIONS:**

Although most pregnancies proceed without complications, the subsequent pregnancies of women with a prior maternal IS are high-risk pregnancies that require careful planning and surveillance. They are frequently complicated with diabetes and hypertensive disorders of pregnancy, and the recurrence of IS or transient ischemic attack is notable.

Maternal ischemic stroke (IS) is a rare complication of pregnancy and may cause significant morbidity and mortality. The incidence of maternal IS has been estimated as 12.2 per 100 000 pregnancies,^1^ and it has been found to be increasing in recent years.^2–4^ The recurrence rate of all maternal stroke subtypes in subsequent pregnancies has recently been estimated at 1.0% to 5.5%.^5,6^ The secondary prevention of IS during subsequent pregnancies is complicated by the risk of fetal adverse events of most evidence-based medications, such as clopidogrel, warfarin and direct oral anticoagulants, statins, and some antihypertensive drugs. Low-dose aspirin (ASA) or low-molecular weight heparins (LMWH) are recommended after individual consideration as a secondary preventive medication of IS during subsequent pregnancies and the postpartum period, as well as specific antihypertensive medications.^7,8^

In our prior study, most women with maternal IS recovered well,^9^ and thus might wish to have future pregnancies. A study on young females with IS showed that the lack of subsequent pregnancies after a stroke, despite a wish for pregnancy, was mostly related to concerns about the potential recurrence of strokes, medical advice against pregnancy, or a residual deficit.^10^ A limited number of studies address the recurrence of IS or other complications in subsequent pregnancies in patients with a history of maternal IS, and none of them solely focus on patients with maternal IS.^5,6,10^ Data on the subsequent pregnancies of patients with maternal IS are needed for adequate preconception counseling.

We collected a nationwide cohort of patients with IS during pregnancy or puerperium in Finland between 1987 and 2016. We aimed to study the recurrence of IS and transient ischemic attack (TIA), as well as other maternal and neonatal complications in subsequent pregnancies. The objectives were to identify the recurrence of IS and TIA in the subsequent pregnancies of patients with maternal IS, and to compare whether the course of subsequent pregnancies and their maternal and neonatal complications are similar between patients with maternal IS and women with no history of a maternal stroke. We also assessed the use of secondary prevention and whether the women were under high-risk pregnancy monitoring during subsequent pregnancies.

## Methods

This article follows the Strengthening the Reporting of Observational Studies in Epidemiology (STROBE) reporting guidelines.^11^

### Data Availability

Because the data collected for this study contains potentially identifying and sensitive patient information, the data cannot be shared in open data depositories. Deidentified aggregated data that support the findings of this study can be made available to qualified investigators on reasonable request to the corresponding author.

### Study Design and Patient Identification From the National Registers

We conducted a retrospective and population-based nationwide study of ISs associated with pregnancy and postpartum in Finland. Women diagnosed with IS during pregnancy or the postpartum period up to 12 weeks after delivery in Finland during the years 1987 to 2016 were identified from national health care registers: the Medical Birth Register (MBR),^12^ Hospital Discharge Register (HDR),^13^ and the Cause of Death Register. The MBR includes all women in Finland with a pregnancy resulting in delivery (live births and stillbirths with gestational age ≥22+0 weeks or with birthweight ≥500 g) since 1987. All inpatient hospitalizations since 1967 and all outpatient clinic visits since 1998 in Finland are registered in the HDR. Disease and procedure codes indicating a stroke or its treatment in the HDR from 9 months (270 days) before and up to 3 months (90 days) after the delivery date in the MBR were used to identify the patients (Table S1). A comprehensive search strategy was used to identify all possible cases.

### Data Collection

The medical records of patients were obtained from the health care facilities where the disease or procedure codes indicating maternal IS were coded. The records were acquired until the end of 2016 in the specialties of neurology, gynecology, neurosurgery, internal medicine, radiology, and laboratory medicine. Clinical data were collected from medical records. The diagnosis of IS and its temporal connection to pregnancy were verified from the medical records by stroke neurologists. The diagnosis of IS was based on clinical findings and radiographic features. Solely, the IS cases of arterial origin were included. We excluded the patients who did not have IS during pregnancy or the postpartum period and recorded the reasons for exclusion, which have been specified previously.^3^

Patients with maternal IS who died within a year of the stroke (n=5) and their controls (n=15) were excluded from the analyses. All subsequent deliveries (live births and stillbirths) of patients with maternal IS and controls were obtained from the MBR. As the register data on subsequent pregnancies were not available for 2 patients with maternal IS, they were excluded from the analyses. The recurrence of maternal stroke and TIA in subsequent pregnancies was collected from the HDR with corresponding *International Classification of Diseases* codes, and the diagnoses were verified from the patient records by stroke neurologists. The demographics of index pregnancy, and maternal and neonatal complications in the subsequent pregnancies were collected from the MBR and supplemented with data from the HDR with corresponding International Classification of Diseases codes during pregnancy and up to 12 weeks postpartum. Additionally, subsequent spontaneous and induced abortions were collected from the HDR with corresponding International Classification of Diseases codes. The diagnostic codes used are described in Table S2. The completeness and accuracy of MBR and HDR are rated as good to satisfactory, and the coverage of the MBR is estimated to approach 100%.^14,15^

Secondary preventive medications and follow-up visits in a specialized health care maternity clinic were collected from the patient records. In Finland, all pregnant women receive antenatal care through public primary health care maternity clinics, with visits provided free of charge. Women with a history of IS are typically referred to a public specialized health care maternity clinic, where they are counseled by an obstetrician, and if needed, referred to a neurologist.

Data were collected from the index pregnancy until the end of 2016. Additional information on the collection and completeness of data is available in Supplemental Methods.

### Definitions

IS was defined as brain cell death attributable to ischemia, based on pathological, imaging, or other objective evidence of a cerebral focal ischemic injury in a defined vascular distribution; or clinical evidence of a cerebral focal ischemic injury based on symptoms persisting ≥24 hours or until death, and other causes excluded.^16^ The causes of ISs were defined using the TOAST (Trial of ORG 10172 in Acute Stroke Treatment) criteria.^17^ The functional outcome was measured based on the modified Rankin Scale at 3 months and at the end of the follow-up.^18^ A good functional outcome was defined as a modified Rankin Scale score of 0 to 2. Pregnancy was defined as an event starting at conception and the postpartum period lasting until 12 weeks (84 days) after the delivery. Maternal complications included any diabetes during pregnancy, postpartum hemorrhage, placental abruption, and hypertensive disorders of pregnancy (HDP), including chronic hypertension, gestational hypertension, preeclampsia, eclampsia, and hemolysis, elevated liver enzymes syndrome, and low platelets syndrome. Neonatal complications included perinatal mortality, prematurity (born <37+0 weeks of gestation), low-birth weight (<2500 g), 1-minute Apgar score of <7, and child hospitalization at 7 days of age. Perinatal mortality was defined as a death from 22 weeks of gestation until the age of 1 week.

### Case-Control Analysis

For a nested case-control analysis, 3 controls were identified from the MBR for each IS case and matched by delivery year, age, parity, and geographic area. Controls were identified from the same cohort of all women who delivered in Finland between 1987 and 2016 (n=1773 728) as the cases. Controls were pregnant women without a pregnancy-related stroke during the study period. No controls for 1 case and only 1 control for another case were available. Only the register data from the HDR and MBR were used in the case-control analyses due to the unavailability of medical records for the controls. Cases and controls were compared by the demographics, mode of delivery, number of subsequent pregnancies, induced and spontaneous abortions, as well as maternal and neonatal outcomes and complications in subsequent pregnancies.

In the sensitivity analyses, we compared patients with maternal IS who had subsequent pregnancies with those who did not. They were compared by age, parity, risk factors of IS, cause by TOAST classification, and modified Rankin Scale at 3 months and at the end of the follow-up. These data were obtained from patient records.

### Outcomes

The primary outcomes were the recurrence of IS and TIA in subsequent pregnancies of women with a history of maternal IS. The secondary outcomes were (1) the maternal and neonatal complications in subsequent pregnancies of patients with maternal IS and controls, (2) the number of subsequent pregnancies, deliveries, and spontaneous and induced abortions of patients with maternal IS and the controls, (3) the use of secondary preventive drugs during subsequent pregnancies: antithrombotic, diabetic and antihypertensive medications, and (4) consultation and a specialized maternity care follow-up visit due to a high-risk pregnancy.

### Statistical Methods

The data were presented as numbers and percentages for categorical variables and the mean and SD or median and interquartile range for continuous or ordinal variables. To test for differences between groups, the χ^2^ test or Fisher exact test was used for categorical variables, and the *t* test or Mann-Whitney *U* test for continuous variables. A *P*<0.05 was considered statistically significant. Binary logistic regression was used to calculate age-adjusted odds ratios (aOR) and 95% CIs. Generalized estimating equations logistic regressions were used to calculate aORs and CIs for the complications and outcomes of subsequent pregnancies. In the case-control study, unconditional logistic regression analysis was used since our data matched on a few demographic variables, and are loose-matching data. Statistical analyses were performed with SPSS Statistics, version 29.

### Ethical Approval

The study has been approved by the Ethics committee of Helsinki University Central Hospital (HUS/2228/2016 13.12.2016), THL Finnish Institute of Health and Welfare (THL/750/5.05.00/2017), and Statistics Finland (TK-53-783-17, TK-53-591-20). Findata, the Finnish Social and Health Data Permit Authority, has granted a permit for the secondary use of these data until December 31, 2033 (THL/1784/14.06.00/2023). Findata was responsible for ensuring the anonymity of the results. According to Findata regulations, to ensure anonymity, a minimum frequency of 3 was used when reporting frequencies. A minimum frequency of 3 can be used if the study includes a very small target group, the results are necessary to report, and identifying patients is not possible. The EU and Finnish data protection legislation allows the use of register data in scientific research without a written patient consent.

## Results

### Maternal IS Cohort and Controls

From 1987 to 2016, there were 1773 738 deliveries, 1792 791 live births, and 6799 stillbirths in the MBR. In total, there were 97 patients with maternal IS. Data on the subsequent pregnancies were available for 90 patients with maternal IS after excluding 5 patients who died within 1 year of IS and 2 patients with no follow-up data. Patient records were available for all. In total, there were 265 controls with register data available on subsequent pregnancies after excluding 15 controls of the patients who died within 1 year of the index IS. The median time from the index delivery until the end of the follow-up was 11.6 years (interquartile range, 4.8–20.7 years). The flowchart of the maternal IS cohort and controls is presented in the Figure. The distribution of cases by 5-year time periods is presented in Figure S1.

**Figure.**
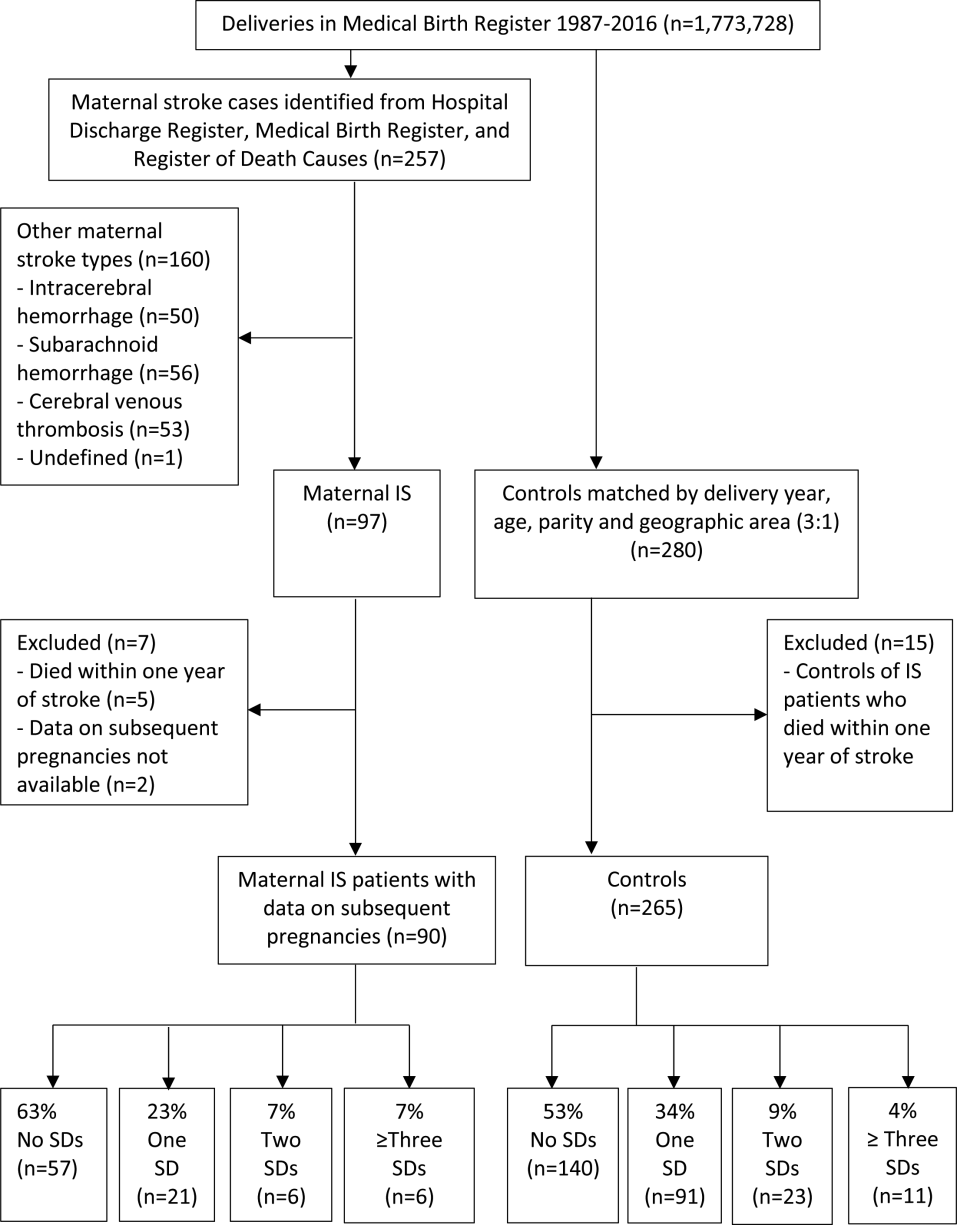
**Flowchart of the maternal ischemic stroke (IS) cohort and controls.** SD indicates subsequent delivery/deliveries.

### Demographics and the Numbers of Subsequent Pregnancies

The clinical features during the index pregnancy and the number of subsequent pregnancies in patients with maternal IS and controls are presented in Table 1. The clinical features during the index pregnancy in patients with maternal IS and controls were similar regarding age and most traditional risk factors of stroke (obesity, smoking, dyslipidemia, and diabetes). During the index pregnancy, patients with maternal IS more frequently had migraines (aOR, 36.74 [95% CI, 4.67–289.0]), HDP (aOR, 4.07 [95% CI, 2.01–8.23]), and cesarean sections (aOR, 3.13 [95% CI, 1.83–5.36]).

**Table 1.**
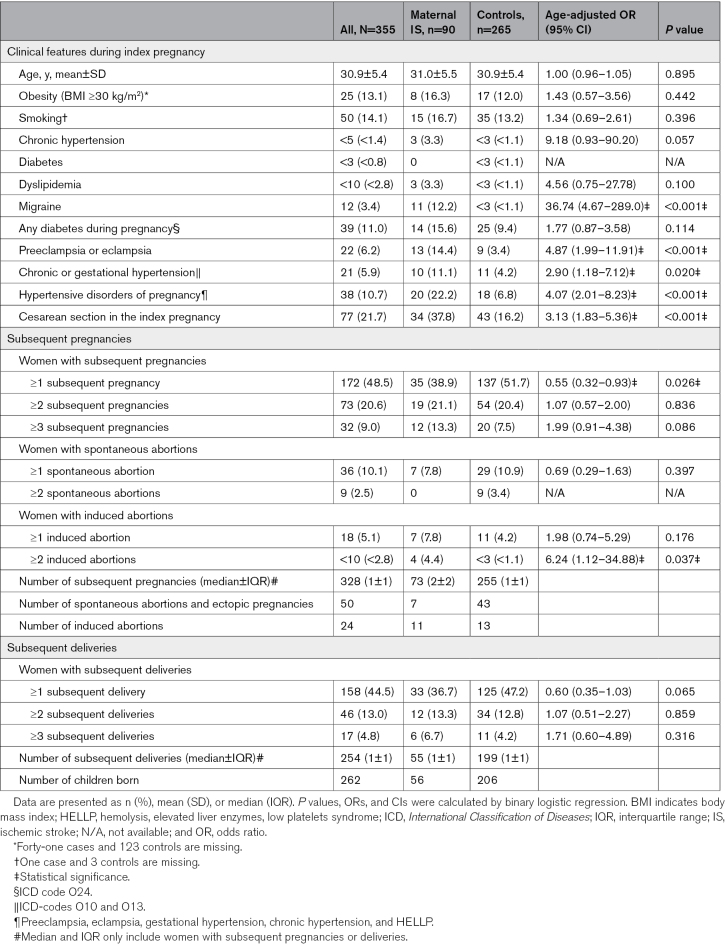
Demographics and Subsequent Pregnancies of the Patients With Maternal IS and Controls

During the follow-up, 35 (38.9%) of 90 patients with maternal IS had subsequent pregnancies and 33 (36.7%) had subsequent deliveries (Figure). There were 265 controls, of which 137 (51.7%) had subsequent pregnancies and 135 (47.2%) had subsequent deliveries. Patients with maternal IS had less frequently had at least 1 subsequent pregnancy compared with controls (aOR, 0.55 [95% CI, 0.32–0.93]). The distribution of cases and controls with and without subsequent pregnancies by 5-year time periods is presented in Figure S2. Furthermore, patients with maternal IS more commonly had multiple induced abortions than controls (aOR, 6.24 [95% CI, 1.12–34.88]). Four patients with maternal IS had 2 induced abortions, of which 87.5% were performed for social reasons. The indications for multiple induced abortions included the fear of pregnancy complications or stroke recurrence in 3 of 4 women. All women with multiple induced abortions had good functional outcomes during the follow-up period. According to patient records, none were advised by health care professionals to undergo an induced abortion due to their prior stroke. There were no statistically significant differences in the proportions of women having at least 1 subsequent delivery or spontaneous abortion. The median time between the index delivery and the first subsequent delivery was 3.3 years (interquartile range, 3.2 years) in patients with maternal IS and 3.4 years (interquartile range, 4.1 years) in controls.

### Secondary Prevention and Follow-Up in Patients With Maternal IS With Subsequent Deliveries

The secondary preventive medication and follow-up of women with a prior maternal IS with subsequent deliveries are presented in Table 2. Antithrombotic medication was used by most (84.8%) before the first subsequent pregnancy. The use of other secondary preventive medication before the first subsequent pregnancy was rare, with <3 patients using antilipid and none using antihypertensive or diabetic medication. In the first, second, and third subsequent pregnancies, antithrombotic medication was used by 87.9%, 83.3%, and 66.7%, respectively. ASA, solely or combined with another antithrombotic, was used by 66.7% during the first and second subsequent pregnancies and by 50.0% in the third subsequent pregnancy. Antithrombotic medication was continued for at least 6 weeks postpartum by 72.7% in the first subsequent pregnancy, decreasing to 66.7% in the third subsequent pregnancy.

**Table 2.**
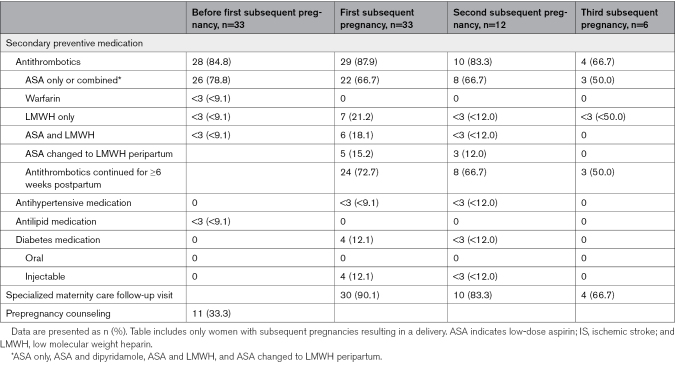
Secondary Preventive Medication and Follow-Up of the Patients With Maternal IS With Subsequent Pregnancies

Three patients did not receive antithrombotic medication in any of the subsequent pregnancies: 2 of them had specialized maternity care visits but secondary preventive medication was not considered in the patient records, and 1 was not recommended to use any medication because the IS cause was a local intracranial artery dissection. There were no patient records of the subsequent pregnancy for 1 patient, but no medication was started after the initial IS, since symptoms were mild. Additionally, 1 patient did not have antithrombotic medication in the second and third subsequent pregnancies because of refusal to use them.

In total, 90.1% of women attended a specialized maternity care follow-up visit due to a prior stroke in the first subsequent pregnancy, and secondary preventive medication was considered in all these patients. The majority (96.7%) had multiple follow-up visits. Additionally, one-third of patients with maternal IS with subsequent deliveries received prepregnancy counseling, and 2 women (6.1%) were advised against future pregnancies due to their prior stroke or frequent complications in previous pregnancies. The distribution of antithrombotic treatment and follow-up during the first subsequent pregnancy by 5-year time periods is presented in Figure S3. A greater proportion of women attended specialized maternity care follow-up visits over time (*P*=0.051), although this did not reach statistical significance. No statistically significant differences were observed in the use of antithrombotics (*P*=0.112) or in the continuation of antithrombotic therapy for at least 6 weeks postpartum (*P*=0.319).

### The Recurrence of IS and TIA

Recurrent ISs or TIAs in subsequent pregnancies occurred in 3 patients (8.6%): 2 had a recurrent IS (5.7%) and 1 had a TIA (2.9%). There were no other stroke types in subsequent pregnancies. Both ISs occurred in the first week of the postpartum period. According to TOAST, the causes of the ISs were undetermined with a negative diagnostic evaluation and other determined, specifically moyamoya disease. Another patient had a TIA in the third trimester of the pregnancy. One patient used ASA and LMWH, 1 used LMWH, and 1 had no medication during the recurrent IS/TIA. Specialized maternity care visits were attended by all of them. One patient with recurrent IS/TIA had HDP in both the index and subsequent pregnancy, whereas 2 patients had no HDPs in either the index or subsequent pregnancies. The median time from the index stroke to recurrent stroke or TIA was 4.5 years, and to recurrent stroke alone, 4.8 years. The events occurred during the first, second, and third subsequent pregnancies.

During the follow-up period (until the end of 2016), there were 3 (3.3%) recurrent strokes and 1 (1.1%) TIA that occurred outside of subsequent pregnancies among women with a history of maternal IS who survived beyond 1 year and had available data (n=90). This corresponds to a total stroke recurrence rate of 5.6% (n=5), and a combined stroke/TIA recurrence rate of 7.8% (n=7) during follow-up. Patients who experienced a recurrent stroke or TIA outside of pregnancy were treated with either ASA or warfarin as an antithrombotic therapy. Half of these patients with recurrent stroke/TIA not related to pregnancy had no subsequent pregnancies, whereas the other half had 1. Among the controls, no strokes and <3 TIAs occurred outside of subsequent pregnancies during the follow-up period.

### Other Complications and Outcomes in Subsequent Pregnancies

The complications and outcomes in subsequent pregnancies of the maternal IS patients compared with controls are presented in Table 3. In subsequent pregnancies, patients with maternal IS more commonly had any diabetes during pregnancy than controls (29.1% versus 13.6%; aOR, 2.77 [95% CI, 1.17–6.59]). Patients with maternal IS more frequently had HDP than controls (12.7% versus 4.5%; aOR, 3.57 [95% CI, 1.02–12.51]). In the first subsequent pregnancy, preeclampsia or eclampsia (*P*=0.043) and HDP (*P*=0.035) were more common in women with a prior maternal IS (Table S3). There were no statistically significant differences in other maternal complications, but women with prior maternal IS had more frequently any maternal complication in subsequent pregnancies than controls (38.2% versus 18.6%; aOR, 3.08 [95% CI, 1.39–6.83]). After adjusting for age and HDP in the index pregnancy, the statistical significancy for HDPs in subsequent pregnancies was lost (aOR, 2.50 [95% CI, 0.83–7.54]). However, the association remained significant for any maternal complication (aOR, 2.75 [95% CI, 1.25–6.07]).

**Table 3.**
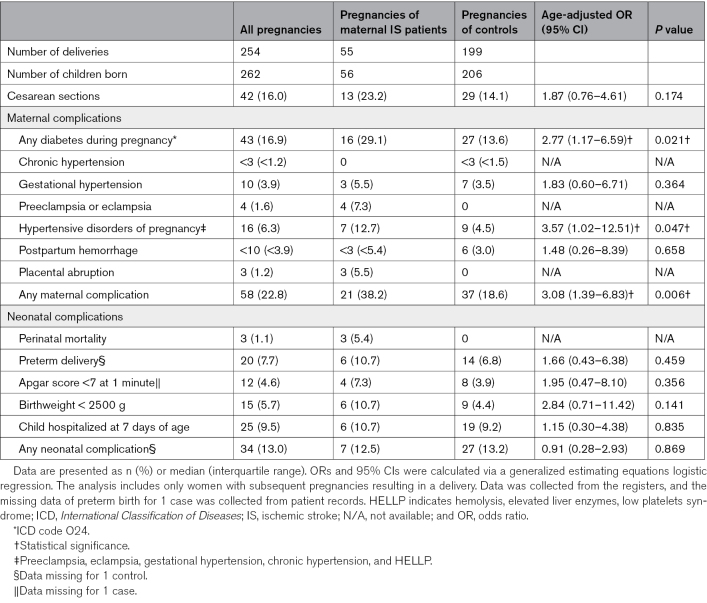
Complications and Outcomes in Subsequent Pregnancies of the Patients With Maternal IS (n=33) and Controls (n=125)

Patients with maternal IS had 3 (5.4%) perinatal deaths in subsequent pregnancies, whereas the controls had none. All were born extremely preterm, before the 28th week of pregnancy. In the first subsequent pregnancy, perinatal deaths were more common in patients with maternal IS compared with controls (*P*=0.042). No stillbirths were recorded. Regarding other neonatal outcomes and complications, there were no statistically significant differences in the proportions of preterm deliveries, low-birth weight, Apgar score <7 at 1 minute, or child hospitalization at 7 days of age.

### Comparison of Patients With Maternal IS With or Without Subsequent Pregnancies

The comparison of clinical characteristics of patients with maternal IS with at least 1 subsequent pregnancy and no subsequent pregnancies are presented in Table S4. The patients with maternal IS with subsequent pregnancies were younger (median, 28.5 versus 32.2 years; *P*<0.001) and more frequently had an undetermined cause of IS via TOAST (71.4% versus 50.9%; *P*=0.027) than those without subsequent pregnancies. Moreover, patients with subsequent pregnancies more commonly had good functional outcomes (modified Rankin Scale score, 0–2) at 3 months (97.1% versus 81.8%; *P*=0.045) and the end of the follow-up (100.0% versus 85.5%; *P*=0.021). There were no statistically significant differences in other clinical characteristics.

A higher proportion of patients had no subsequent pregnancies when the index stroke occurred between 2012 and 2016; however, this was not significantly different compared with controls (79.2% in cases versus 61.4% in controls; *P*=0.114). Prepregnancy counseling in specialized health care was provided to 11 women (20.0%) with no subsequent pregnancies, and 2 of them (3.6%) were advised against future pregnancies due to frequent complications in prior pregnancies. Sterilization after the index pregnancy was performed in 12 patients (21.8%) with no subsequent pregnancies. In half of these cases, the decision was influenced by the increased risk of complications in future pregnancies or residual symptoms from the stroke. Among the 43 patients with no subsequent pregnancies and no sterilization, 67.4% had a parity of at least 2 after the index delivery, 27.9% were ≥35 years old at the time of the index delivery, and 4.8% had poor functional outcomes at 3 months.

## Discussion

In this nationwide retrospective cohort study, we examined the recurrence of IS, TIA, and other complications in the subsequent pregnancies of women with a prior maternal IS, as well as the use of secondary prevention in subsequent pregnancies. Three women with a prior maternal IS and subsequent pregnancies had a recurrent maternal IS or TIA. Women with a prior maternal IS more commonly had diabetes during pregnancy and HDP during subsequent pregnancies than controls; although, there were no statistically significant differences in other maternal or neonatal complications. The majority of the women attended a specialized maternity care follow-up visit and used antithrombotic medication during the first subsequent pregnancy, but this declined in subsequent pregnancies. The use of other secondary preventive medications was uncommon both before and during pregnancy. Patients with maternal IS less frequently had at least 1 subsequent pregnancy and more often multiple induced abortions than controls.

Recurrent IS occurred in 5.7% and recurrent IS/TIA in 8.6% of the patients with maternal IS with subsequent pregnancies. Both ISs occurred in the first week of the postpartum period and TIAs in the third trimester of pregnancy. All women had attended a specialized maternity care follow-up visit. During the recurrent IS or TIA, 1 patient used LMWH and ASA, 1 used solely LWMH, and 1 patient had no medication. In a French study, 1.0% of women with a prior maternal stroke and subsequent pregnancies had a recurrent maternal stroke, but none of these occurred in women with prior maternal IS.^6^ In another French study, there were no recurrent strokes in subsequent pregnancies of women with a prior maternal IS or cerebral venous thrombosis.^10^ A literature review found a 2% risk of stroke recurrence in subsequent pregnancies after maternal stroke; however, none of these cases involved women with a history of maternal IS.^19^ The median follow-up time in the French study was 5.5 years,^6^ whereas in other studies, it was either 5 years or not reported.^10,19^ In the French study, only 15% of women had subsequent pregnancies,^6^ and other studies included only 1 to 15 patients.^19^ Compared with these studies, the recurrence in our cohort appears higher despite active surveillance and the use of secondary preventive medication. Possible explanations for this include the longer follow-up period and larger patient sample in our study compared with most previous studies. Additionally, the secondary prevention may not have been optimal in all patients. Stroke/TIA recurrence outside of subsequent pregnancies was slightly less frequent but still notable (4.4%) in our cohort, and this was comparable to the rate reported in the French study (6.5%).^6^

Overall, in our maternal IS cohort, 88% of women used antithrombotics and 73% continued for at least 6 weeks postpartum in the first subsequent pregnancy. Specialized maternity care follow-up visits were attended by 90% in the first subsequent pregnancy and prepregnancy counseling was given to a third of patients with subsequent deliveries. The proportion of women using antithrombotics and attending antenatal follow-ups declined in the second and third subsequent pregnancies. The use of other secondary preventive medication was quite rare in these young fertile-aged women. During the study period, there were no specific recommendations for secondary stroke prevention during pregnancy, and perceptions of the safety of different drugs have also changed, which is reflected in the variation in medication use during the study period (Figure S3). A few guidelines for the secondary prevention of IS during pregnancy and puerperium have been published since 2017,^7,8,20^ but not specifically in case of a prior maternal stroke. It is recommended to make decisions regarding antithrombotic use on an individual basis.^7,8,20^ Low-dose ASA and LMWH are considered safe throughout pregnancy and puerperium, and considering the higher risk of IS during peripartum and early puerperium, an antithrombotic should be continued during the puerperium.^7,8,20^ Prepregnancy counseling and planning secondary prevention, is recommended for everyone with a history of stroke.^8^ Future pregnancies are not contraindicated in women with a prior stroke, according to the available data.^20^

The proportion of women having at least 1 subsequent pregnancy was lower in patients with maternal IS, and they more commonly had multiple induced abortions, for which the reasons included the fear of pregnancy complications or stroke recurrence. Increased risk of pregnancy complications or stroke residual symptoms also affected the decision of sterilization. Patients with subsequent pregnancies had better functional outcomes, were younger, and more commonly had an undetermined cause of stroke than those with no subsequent pregnancies. Prepregnancy counseling in patients with no subsequent pregnancies was uncommon, and few were advised against subsequent pregnancies. Potentially, a larger proportion of women with prior maternal IS avoided subsequent pregnancies, which appears to be affected by recovery from stroke, comorbidities, and fear of complications in subsequent pregnancies, but also by age and parity. Problems in finding suitable contraception might also affect the number of induced abortions, since combined oral contraceptives are not recommended in women with a previous stroke.^20^ Despite counseling and surveillance becoming more common over time, the proportion of patients having subsequent pregnancies remained low. The counseling may not fully address factors that influence decision-making regarding subsequent pregnancies. These include patient-centered factors, such as fear of pregnancy complications, uncertainty about prognosis, and potential stroke sequelae, like poststroke depression and fatigue, and health care professional-related factors, such as incorrect or insufficient information about the safety of pregnancy. To support decision-making, counseling should address the risks associated with future pregnancies, provide information on surveillance and secondary prevention, and include a discussion of any specific fears.

In subsequent pregnancies, women with a prior maternal IS had almost 3-fold odds for having any diabetes during pregnancy and almost 4-fold odds for HDP compared with controls. After adjusting for HDP at the index pregnancy, the observed difference was no longer statistically significant. This suggests that the accumulation of stroke risk factors and comorbidities likely plays a more substantial role in the increased frequency of complications in subsequent pregnancies, rather than the stroke itself being the direct cause. There were no statistically significant differences in other maternal or neonatal complications. However, among maternal IS patients, there were 3 perinatal deaths (5.4%) compared with none in controls. Previous studies on complications and outcomes of the subsequent pregnancies have examined mostly women with a prior nonpregnancy-related IS or TIA. In a Dutch study, there was a higher prevalence of HDP, hemolysis, elevated liver enzymes, and low platelets syndrome, and early preterm delivery in subsequent pregnancies of young women with a prior IS or TIA when compared with the general population.^21^ In a Finnish study, there was a higher incidence of hospital admissions during pregnancy and perinatal deaths after IS compared with controls with no history of a stroke, but there were no differences in other neonatal complications.^22^

There are limitations in this study. The cohort size is small due to the rareness of maternal ISs and the small size of the Finnish population. The small number of patients and the rather small number of subsequent pregnancies should be noted when interpreting the results of statistical analyses. The Finnish population is relatively homogenous, and the health care system is heavily subsidized, which might lead to results not being applicable to all populations. The index pregnancies that resulted in induced or spontaneous abortions before 22 weeks of pregnancy were not included, which might lead to an underestimation of the number of maternal IS cases. However, the data on IS or TIA recurrence in subsequent pregnancies was also collected before the 22nd gestational week. Although the median follow-up time was rather long, it was short for patients who had the initial maternal IS in the late years of the study. There are several limitations related to the retrospective nature of register-based data collection. Detection bias may have influenced the identification of risk factors, potentially leading to the underestimation of several risk factors among controls compared with IS cases. In the earlier years of the study period, diagnostic coding may have been less complete, which would affect data accuracy. Additionally, diagnostic practices and treatment approaches for IS and related comorbidities have evolved over the study period, which may have influenced the results. As the MBR and HDR only include the data of subsequent deliveries in Finland, we do not have the data of patients who had subsequent deliveries or abortions abroad. The strengths of this study include the nationwide nature, long sampling period, and follow-up time, as well as the verification of the IS diagnoses from patient records.

To conclude, women with a prior maternal IS were less frequently had at least 1 subsequent pregnancy, and the subsequent pregnancies were more commonly complicated with diabetes during pregnancy and HDP. The recurrence of IS or TIA in subsequent pregnancies was notable, and higher than reported in previous studies. Even though most pregnancies were successful, the increased risk of complications highlights the need for prepregnancy counseling and close monitoring of subsequent pregnancies. Maternal complications could likely be reduced through screening for HDPs and other stroke risk factors, regular follow-ups, and the initiation of appropriate secondary prevention. The use of antithrombotics should always be considered and, if indicated, continued for at least 6 weeks postpartum.

## ARTICLE INFORMATION

### Sources of Funding

The following fundings were received: competitive state research funding for the responsibility area of the Helsinki and Uusimaa Hospital District in 2017 to 2018, research funding from the Neurocenter of Helsinki University Hospital in 2019 to 2020, and a personal grant for Dr Richardt from Aarne Koskelo Foundation, Finnish Brain Foundation, and Maire Taponen Foundation, and a personal grant for Dr Aarnio from Maire Taponen Foundation.

### Disclosures

Dr Laivuori reports grants from Finska Läkaresällskapet, Sydäntutkimussäätiö, Tampere University Hospital, and Academy of Finland. The other authors report no conflicts.

### Supplemental Material

Supplemental Methods

Tables S1–S4

Figures S1–S3

STROBE Checklist

## Supplementary Material


